# Automated surgical workflow recognition in privacy-preserving depth videos of the operating room

**DOI:** 10.1007/s00464-025-12031-6

**Published:** 2025-08-06

**Authors:** Beerend G. A. Gerats, Jelmer M. Wolterink, Ivo A. M. J. Broeders

**Affiliations:** 1 Surgery Department, Meander Medical Center, Maatweg 3, Amersfoort, 3813 TZ Utrecht The Netherlands; 2 Robotics and Mechatronics, University of Twente, Drienerlolaan 5, Enschede, 7522 NB Overijssel The Netherlands; 3 Department of Applied Mathematics & Technical Medical Center, University of Twente, Drienerlolaan 5, Enschede, 7522 NB Overijssel The Netherlands

**Keywords:** Operating room efficiency, Surgical workflow recognition, Depth videos

## Abstract

**Background:**

Efficient operating room (OR) workflows have the potential to reduce delays and cancellations, shorten patient waiting lists, and improve satisfaction among patients and staff. Insights for OR efficiency can be extracted from the registration and timing of workflow steps. However, manual registration of these steps is often unreliable. Therefore, we propose to recognize the OR workflow automatically in videos from overhead depth cameras using deep learning. In contrast to regular cameras, depth cameras do not capture fine video details that permit identification of the people recorded. Hence, the privacy of patients and staff is preserved.

**Methods:**

We gathered a video dataset of 21 laparoscopic surgeries captured by three depth cameras positioned in different corners of the OR. The procedures were annotated with four phases describing the OR workflow, i.e., *turnover*, *anesthesia*, *surgery*, and *wrap-up*. We performed an extensive analysis with spatial and temporal deep learning models, including a comparison between multi- and single-view camera setups, and contrasting post-operative with real-time predictions. Along with standard metrics for workflow recognition, we introduce a new evaluation metric that reflects the error in estimated phase duration.

**Results:**

The best-performing model, ASFormer, recognized operative phases with 99.7% mean average precision (mAP), enabling the estimation of phase duration with a mean absolute error of 35 seconds. The best-performing spatial model resulted in 89.7% mAP, indicating the importance of temporal modeling. We also found that the three cameras could be replaced by a single camera, with 98.8% mAP, although performance depends on the camera location in the OR. Additionally, we found that real-time prediction is feasible but underperforms with respect to post-operative analysis (94.3% mAP).

**Conclusions:**

Automated OR workflow recognition is possible using existing deep learning techniques based on single- and multi-camera setups. The use of privacy-preserving depth videos and a reasonably low phase duration estimation error could have positive implications for practical use.

**Supplementary Information:**

The online version contains supplementary material available at 10.1007/s00464-025-12031-6.

Due to the aging population and personnel shortages, the demand for staffed operating room (OR) facilities is increasing every year [1]. These developments make OR efficiency an essential topic for hospital management, aiming to optimize procedure scheduling, avoid delays and cancellations, and decrease long waiting lists for patients [2]. For the optimization of OR efficiency, it is key to find insights into how the ORs are used over time. In various hospitals, insights such as turnover time and schedule delays are generated by manual registration and timing of steps in the OR workflow. However, these registrations are often unreliable due to human errors, lack of standardization, and limited time to perform the registrations [3]. In this research, we propose to automate workflow recognition in videos from overhead cameras to obtain objective, standardized, and reliable registrations.

Automatic recognition of actions in OR videos has been applied to orthopedic surgery, although the actions were limited to those performed by the surgeon [4, 5]. Other research was directed toward robot-assisted surgery, with the introduction of an extensive dataset by Shargi et al*.* [6]. Their incentive was to recognize ten activities, half of which described the control of the surgical robot. In follow-up research, this dataset was extended with more videos and used to improve action recognition methods [7–9], to explore training with fewer annotated videos [10, 11], and to increase generalizability across multiple ORs [12]. In this paper, we shift focus to laparoscopic surgery, as robot-assisted surgery is only a small fraction of all endoscopic procedures. Bastian et al*.* [13] could automatically identify eight operative phases in laparoscopic porcine surgeries. In contrast to their research, we explore the use of a wide range of deep learning models and use data from surgery performed on people.

Although the use of overhead cameras in the OR brings rich data in a relatively cheap manner, it raises privacy concerns for patients and personnel. Most related works make use of full-color or depth combined with intensity videos [4–7, 10, 11, 14, 15], wherein the recorded people are identifiable. To preserve the privacy of patients and staff, we propose to use depth videos only. This data modality is often gathered with a time-of-flight (ToF) sensor and removes the need for error-prone and labor-intensive blurring. Additionally, depth is perceived as most anonymous by the people captured in the video [16] when compared to other forms for de-identification.

This paper explores video-based recognition of phases in non-robotic laparoscopic surgeries. We perform an extensive study with various temporal and non-temporal models, a comparison between multiple and single-camera setups, and including offline (post-operative) and online (per-operative) models. In addition to standard metrics for workflow recognition, we introduce a new evaluation metric that reflects the error in estimated phase duration. This metric is more interpretable with the goal of optimizing OR efficiency in mind. Using the technology presented in this paper, the start and end times of each operative phase can be identified objectively for comparison with scheduled times, while phase durations can be reported for another layer of efficiency insights.

## Materials and methods

### Multi-camera depth video dataset

We recorded 21 laparoscopic surgeries with three Azure Kinect RGB-D cameras (Microsoft Inc., Redmond, WA, USA), each positioned in a different corner of the OR. The recordings were performed over seven days in a single hospital, involving six procedure types: cholecystectomy, appendectomy, diaphragmatic hernia repair, inguinal hernia repair, hernia umbilical repair, and tumor resection. The data collection was approved by the local ethical committee, and all patients and staff agreed to be subject to our study.

**Fig. 1:**
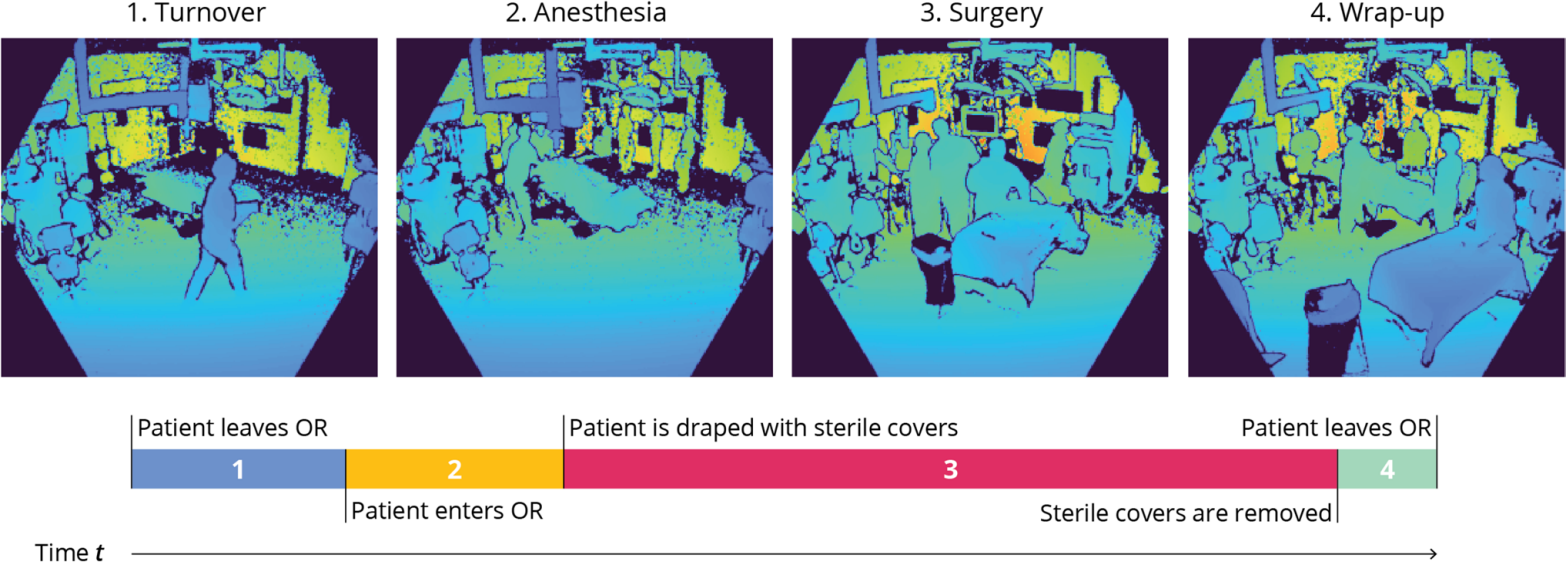
Description of the phase transitions, along with a sample depth frame for each of the four phases. The color of the pixels reflects the distance between the camera and the nearest object

All videos, recorded at one frame-per-second (fps), are manually annotated with four operative phases that describe the OR workflow coarsely: *turnover*, *anesthesia*, *surgery*, and *wrap-up*. A description of the phase transitions is given in Fig. 1 together with an example frame per phase. The number of frames in our dataset and the average duration per phase can be found in Table 1. The phase transitions are selected based on two highly visible events: 1) the patient entering or leaving the OR and 2) the draping or undraping of the patient with sterile covers by the surgical team. As the latter event is a distinct action early in the surgical preparation, this moment indicates that the surgical team has started and the anesthetic preparations are finished.

**Table 1:** Target operative phases with the number and percentage of frames in the dataset together with the average duration

Phase	# frames	% of total	Average phase duration
Turnover	16,621	16.4	9 m 53 s (± 8 m 13 s)
Anesthesia	16,931	16.7	13 m 26 s (± 5 m 21 s)
Surgery	60,187	59.3	47 m 46 s (± 23 m 10 s)
Wrap-up	7712	7.6	6 m 7 s (± 2 m 10 s)

### Deep learning methods for workflow recognition

We perform an extensive evaluation of deep learning techniques for workflow recognition, including two types of spatial models (ResNet [17] and Vision Transformer [18]) and three types of spatio-temporal models (MS-TCN++ [19], ASFormer [20], and DiffAct [21]). The first two models use spatial features only, meaning that the recognition of a phase in a frame is based solely on information from that single frame. In contrast, the three temporal models incorporate information extracted from earlier and later frames. In this way, initial single-frame errors can be corrected using a global whole-video context. For the spatio-temporal models, we use the best-performing spatial model as a feature extractor.

**Fig. 2:**
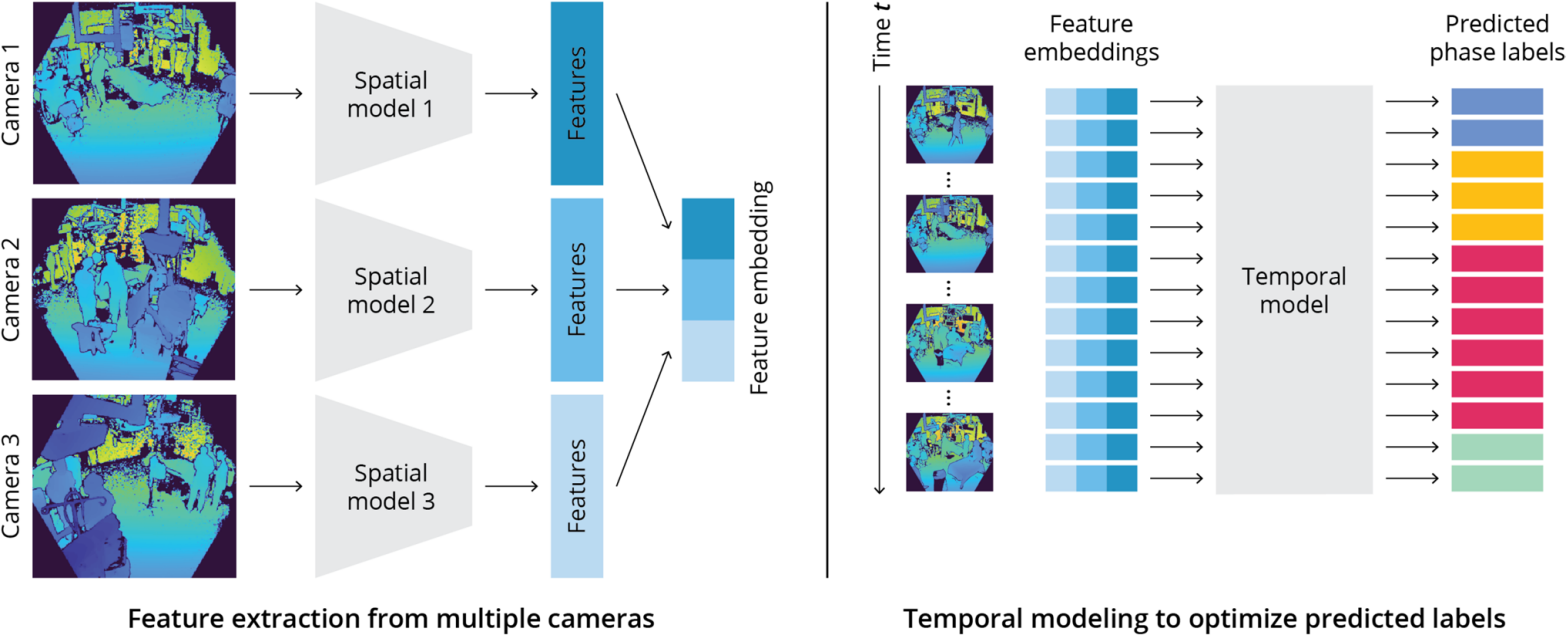
Overview of the data pipeline from multi-view depth video to predicted phase labels. Features are extracted per camera view and combined into a single feature embedding. In a second step, features are exchanged between all time points to optimize the model predictions with global context

The difference between spatial and spatio-temporal models is highlighted in Fig. 2, which shows our data pipeline from a multi-view depth video to predicted phase labels. First, features are extracted for each camera in parallel by training a different model per viewing angle. The models have the same architecture but do not share their weights during training. In this way, each model is specialized for one camera. The features from all views are concatenated to obtain the feature embedding that describes all angles at a single point in time. When only a spatial model is used, the feature embedding is used directly to predict the phase label. With the temporal models, the feature embeddings at all time points are collected, features are exchanged, and a global context is created that treats the video as a whole. The output of a temporal model is a predicted phase label for each time point. In our experiments, we evaluate the performance with two- and single-camera setups as well. For these setups, the data pipeline is the same, although one or two feature extractors are turned off for the excluded cameras. We use a dimensionality of 512 for the extracted features of each camera, meaning that the feature embedding for a single frame has a size of 1536 for three cameras, 1024 for two cameras, and 512 for one camera.

### Online and offline models

For most OR efficiency insights, it is possible to record OR videos first and process the videos post-operatively (“offline”). However, for some use cases, it is important that workflow recognition is performed in real-time (“online”). For an automated overview of the status of all ORs in the hospital, our models should be able to make predictions instantly. Importantly, the selected spatio-temporal models are designed such that the phase recognized in one frame depends on information from earlier and later frames. In the online setting, information from later frames is not available. Therefore, we have adapted the MS-TCN++ [19] and ASFormer [20] such that the timeline segmentation depends on past information only. We followed the adaptions suggested in earlier work by Czempiel et al*.* [22] and Zhang et al*.* [23] for MS-TCN++ and ASFormer, respectively.

### Model and hardware details

We provide a brief introduction to the models used here, along with the hardware used for training. ResNet [17] is a convolutional neural network that is routinely used for a wide range of computer vision tasks and domains. Vision Transformer [18] gained popularity for its ability to scale easily to enormous datasets. MS-TCN++ [19] uses convolutions over the temporal dimension to mix features from neighboring frames. ASFormer [20] uses the Transformer architecture for this purpose instead. Both models have been used for the recognition of phases in laparoscopic video [22, 23]. Last, DiffAct [21] uses a different architecture based on the infusion of noise to learn a typical sequence of workflow steps.

All models are trained on a local workstation with a single NVIDIA 1080Ti GPU. Training times for the spatial models varied between 30 and 120 min for ResNet and ViT, respectively. Using pre-computed spatial features, another 5 to 60 min is required to train the spatio-temporal model, depending on the model type. The depth video recordings took an approximate of 150 GB of data storage. Further details on the deep learning models, including hyperparameters, are given in Supplementary Material.

### Evaluation setup

To evaluate the performance of the selected models on the depth video dataset, we selected the metrics that are standard for workflow recognition: mean average precision (mAP), frame-wise accuracy, edit score, and segmental F1 score (F1@*k*) [24]. The mAP score reflects an average precision over multiple recall levels, and the frame-wise accuracy is the number of frames with a correctly identified phase over the total number of frames. The edit score measures the correctness of the order of phase segments by counting the number of insertions, deletions, and substitutions that are required to transform the predicted sequence into the ground truth sequence. The F1@*k* score measures the intersection-over-union (IoU) between predicted segments and ground truth segments, where a minimal IoU of *k* is treated as a correctly recognized segment. We select three relatively strict values for *k* (50, 75, 95) as insights for OR use can only be reliable when there is a large overlap between predicted and ground truth segments.

We add another metric that is more interpretable for the use case of OR insights, namely “phase duration estimation”. For each procedure, we sum up the time spent on each phase and calculate the absolute error between the estimated durations and the actual durations in the ground truth. We calculate the mean average error (MAE) and the mean error relative to the lengths of the actual times.

Since the dataset is relatively small, all experiments are carried out in sevenfold cross-validation. As the end and start of two consecutive procedures are visually similar, they should not be split into a training and validation set. Thus, we keep all procedures performed on the same day in the same fold to avoid data leakage from the validation to the training set, resulting in seven folds for seven days of recording. The size of the folds varies between one and nine, as the number of procedures is not equal for all recording days. On average, eighteen procedures are used for training and three procedures for validation. Cross-validation ensures that each procedure is part of the validation set once.

## Results

First, we provide the results for workflow recognition with spatial and spatio-temporal models and discuss the performance per action class. Second, we compare the multi-camera results with two-camera and single-camera setups. Last, we show the difference in performance for online and offline models.

### Spatial and spatio-temporal models

**Fig. 3:**
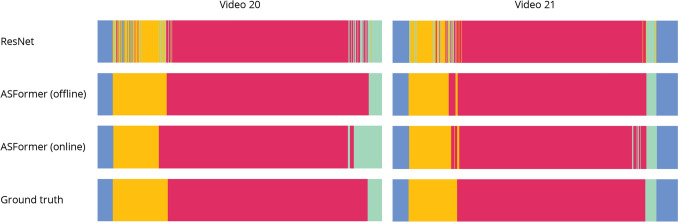
Workflow recognition results for the last two videos in the dataset, for the best-performing spatial model (ResNet) and spatio-temporal model (ASFormer). For the latter, we distinct between offline and online prediction as well. The phase labels are turnover (blue), anesthesia (orange), surgery (red), wrap-up (green)

Table 2 gives the performance scores for various spatial and spatio-temporal models on our dataset. We find that the spatio-temporal models result in more accurate workflow recognition than spatial models, with 89.7% and 99.7% mAP for the best-performing spatial and spatio-temporal model, respectively. The spatial models perform poorly in terms of phase duration estimation, with an MAE of over 10 min for ResNet and over 15 min for Vision Transformers (ViT). In contrast, phase durations are estimated with an MAE of 44 and 35 s for MS-TCN++ and ASFormer, respectively. Figure 3 displays model predictions from ResNet and ASFormer, along with ground truth labels, for the last two videos in the dataset. Where the spatial model has a large amount of noise in the predicted phases, these errors are largely compensated for with the spatio-temporal model. Additionally, we find that ResNet performs superior to ViT. Therefore, we use ResNet as a feature extractor for all spatio-temporal models in this and further experiments. For the spatio-temporal models, we see that DiffAct performs slightly worse than MS-TCN++ and ASFormer.

**Table 2:** Performance metrics for workflow recognition with spatial (top two rows) and spatio-temporal (bottom three rows) models

Model	mAP	Acc	Edit	F1@50	F1@75	F1@95	Duration est	
							MAE	Rel. (%)
ResNet	89.7	71.7	1.0	0.8	0.5	0.3	10 m 6 s	90.9
ViT	51.2	57.7	1.0	0.4	0.3	0.1	15 m 52 s	102.8
MS-TCN++	*99.3*	*98.1*	**99.5**	**99.6**	*94.3*	*72.1*	*44 s*	*5.9*
ASFormer	**99.7**	**98.5**	*96.3*	*96.9*	**95.8**	**76.9**	**35 s**	**3.9**
DiffAct	96.2	96.3	97.1	94.4	90.4	61.8	1 m 13 s	12.2

Best scores are in **bold** and second-best are *italics*. Phase duration estimation is given in absolute time and relative to the phase length

Classification scores per action class can be found in Table 3, where only the results for our best-performing model (ASFormer) are given. The classes *turnover* and *surgery* are identified accurately with F1 scores of 99.3% and 99.0%. Visually, these phases are very distinct due to the absence of the patient (*turnover*) and the surgical team around the operating table (*surgery*). The two other phases are more difficult to distinguish, with F1 scores of 96.8% for both *anesthesia* and *wrap-up*. These phases are visually similar, with people walking around and the patient bed moving to or from the main OR door. Nevertheless, the durations of all phases are estimated with an MAE under a minute.

**Table 3:** Performance metrics for workflow recognition with ASFormer given per class

Class	Precision	Recall	F1	Duration est	
				MAE	Rel. (%)
Turnover	99.6	99.0	99.3	8 s	1.1
Anesthesia	97.7	95.9	96.8	53 s	6.0
Surgery	98.5	99.5	99.0	56 s	1.9
Wrap-up	97.9	95.8	96.8	23 s	6.4

### Single-camera setup

**Fig. 4:**
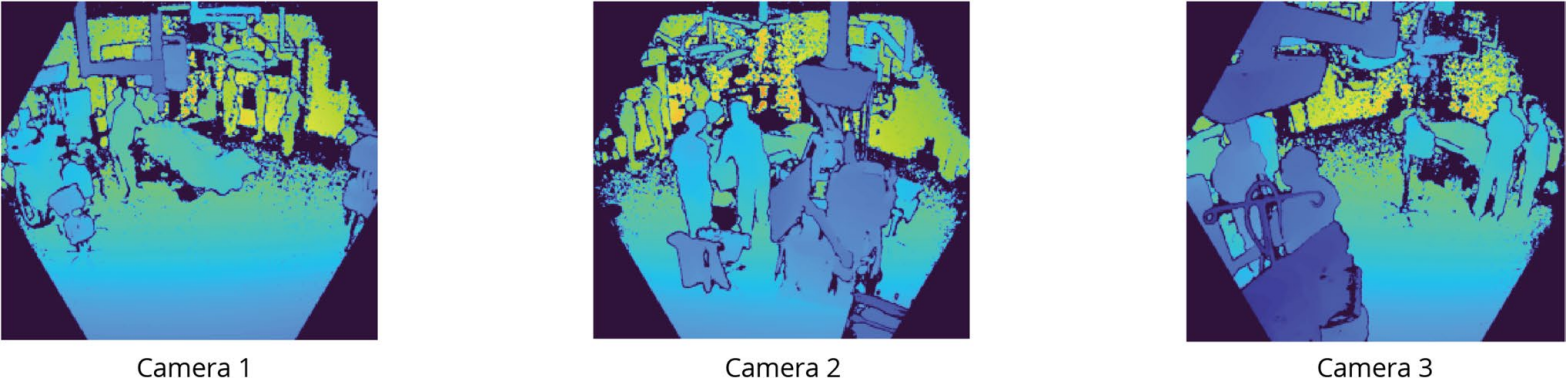
Field of view for all three cameras in the dataset

Multi-camera systems have the advantage of preventing errors from occlusions, as the scene is captured from various viewing points. Occlusions are common in the OR, with surgical lamps, large medical devices, and people potentially obstructing the camera view. However, multiple cameras add complexity and costs to the system as well. In Table 4, we compare workflow recognition performance for multi- and single-camera systems. It can be seen that the performance metrics for a single camera vary largely, between 98.8 and 93.5% mAP for cameras 1 and 2, respectively. Hence, the single-camera performance is heavily dependent on the location of the camera. Figure 4 displays all three camera locations. The best location, camera 1, is directed toward the surgical field and the main door of the OR. Cameras 2 and 3 capture the surgical field only and are partially blocked by medical devices. Using only camera 1, phase durations are estimated with an MAE of 54 s, which is comparable to the MAE of 35 s when using all three cameras.

**Table 4:** Performance metrics for workflow recognition with ASFormer using various cameras as input views

Input views			mAP	Acc	Edit	F1@50	F1@75	F1@95	Duration est	
1	2	3							MAE	Rel. (%)
✓			*98.8*	*97.3*	**98.5**	**97.4**	*92.6*	55.2	*54 s*	*6.9*
	✓		93.5	90.0	75.0	72.3	59.5	38.3	2 m 23 s	20.9
		✓	97.0	93.2	82.9	79.9	75.2	43.5	2 m 28 s	19.8
✓	✓		98.2	96.3	89.3	88.4	85.4	*63.3*	1 m 27 s	11.2
✓		✓	98.1	94.6	83.0	83.5	79.1	55.8	1 m 50 s	13.3
	✓	✓	92.4	87.0	78.0	74.1	67.1	44.0	4 m 42 s	37.2
✓	✓	✓	**99.7**	**98.5**	*96.3*	*96.9*	**95.8**	**76.9**	**35 s**	**3.9**

Best scores are in **bold** and second-best are *italics*

### Online workflow recognition

Where efficiency insights could generally be calculated post-operatively, there is a demand for real-time workflow recognition as well. For example, for a status overview of all ORs in a hospital, it is necessary that the model involved makes instant predictions. Therefore, we compare the performance of offline and online models, where the latter cannot use future information while identifying the action class for each time step. The results are displayed in Table 5 for two temporal models. We find that performance drops from 99.3% to 97.0% mAP for MS-TCN++ and from 99.7% to 94.3% mAP for ASFormer. With an accuracy of around 96%, both online models are correct for a large part of the video. However, the decrease in segmental metrics for the online models indicates flickering action labels. The flickering can be seen in Fig. 3, where the number of phase transitions is larger for the online model than with the offline model. This observation is supported by the performance drop in edit score, which decreases to 47.8% and 74.2% for MS-TCN++ and ASFormer, respectively. Nevertheless, phase durations are estimated with an MAE of 1 min and 17 s, which is still usable in daily practice.

**Table 5:** Performance metrics for workflow recognition for offline and online models

Model	Causal	mAP	Acc	Edit	F1@50	F1@75	F1@95	Duration est	
								MAE	Rel. (%)
MS-TCN++	Offline	99.3	98.1	99.5	99.6	94.3	72.1	44 s	5.9
MS-TCN++	Online	97.0	96.6	47.8	52.4	47.2	34.7	1 m 19 s	8.3
ASFormer	Offline	99.7	98.5	96.3	96.9	95.8	76.9	35 s	3.9
ASFormer	Online	94.3	96.1	74.2	77.1	67.7	37.8	1 m 17 s	12.1

The online models cannot use extracted features from the future and make phase predictions in real-time

## Discussion

In this work, we have explored the use of multi- and single-camera depth videos for automated surgical workflow recognition using deep learning methods. Using a dataset of 21 laparoscopic surgeries, we trained various spatial and spatio-temporal models for the identification of four phases that describe the OR workflow coarsely. We found that spatio-temporal models MS-TCN++ and ASFormer are very successful in workflow recognition, with an mAP of 99.3% and 99.7%, respectively. When the performance is expressed in terms of phase duration estimation, which is more interpretable for efficiency insights, we saw precise estimations with an MAE of 35 to 44 s for these models. In daily practice, such errors are acceptable as the average phase duration, and its variation is a multitude of these numbers. For example, the shortest phase (*wrap-up*) lasts six minutes on average, with a standard deviation of two minutes.

Our study utilizes depth imaging, which, unlike routine cameras, captures anonymous video. Personnel and patients cannot be identified in the videos, making implementation easier under strict privacy regulations. Additionally, anonymous video enables digital processing on hardware outside the hospital. Because a depth camera captures less video detail than a routine camera, one could expect workflow recognition performance to be worse. However, we find that depth cameras and non-anonymous cameras [10, 11, 14] appear to achieve similar performance. Where our method achieves 99.7% mAP on the detection of four events in the operating room workflow, state-of-the-art with routine cameras achieves 92.9% mAP for the detection of eight events [14]. Nevertheless, a direct comparison on a unified dataset is needed to confirm equivalence.

Various efficiency parameters and insights into day-to-day OR use can already be provided with our current model. For example, the scheduled duration for surgery and other operative phases, such as the delivery of anesthesia, can be compared to the duration measured by our system. Such comparisons can isolate procedures that caused program delays, which can be inspected at a deeper level by OR management. Importantly, the duration measurements are not limited to the surgical phase only, i.e., deviations in turnover, anesthesia, and wrap-up are discoverable as well. Similarly, scheduled starting times of procedures could be compared to actual times when the patient has entered the OR. In this way, it is possible to detect program delays as early as possible in an automated manner.

Even though such efficiency parameters can be generated from manual time registrations, our automated system can overcome the errors that generally come with manual registration. These registrations are not always immediately inputted or are not entered until the end of the day, resulting in less accurate registrations than those from an automated system [3]. Additionally, an algorithm can solve for interpersonal differences in definitions of phase transitions. One starts the *anesthesia* phase when the anesthesiologist enters the OR, for example, while the other starts at the anesthetic delivery or even the intubation. An automated system can make the registrations more objective, accurate, and uniform. Nevertheless, our algorithm should be compared to manual time registrations to validate these benefits.

Although a multi-camera system has the advantage of compensating for occlusions, it adds complexity and costs. A single-camera setup is likely to improve the robustness of the system as less hardware needs to be maintained and potential errors in synchronous video recording are avoided. To the best of our knowledge, this study is the first to show that a multi-camera system can be replaced by a single camera at the cost of a small performance decrease. We found that workflow recognition performance decreased with 0.9% mAP, and the MAE for phase duration estimation increased with nineteen seconds, compared to a three-camera setup. However, the performance depends hugely on the location of the camera, as we saw larger errors for cameras not ideally positioned in the OR. We hypothesize that a clear view of the operative field and the main door, where the patient enters and leaves the OR, is essential for good performance.

Besides post-operative insights in OR use, there are demands for real-time action identification. For example, when the start of the *wrap-up* phase is identified, the post-anesthesia care unit (PACU) could receive a warning that a new patient is soon to enter. We compared two temporal models, MS-TCN++ and ASFormer, in both offline (post-operative) and online (real-time) settings. Workflow recognition performance decreased with 5.4% mAP, and the MAE for phase duration estimation increased with 42 s, at worst. Although the outcomes are still reasonable for daily use, more incorrect phase transitions should be expected in the model output. Adding a delay in prediction, together with temporal smoothing or thresholding, could result in more robust online workflow recognition.

Although various endoscopic procedure types are included in the dataset, our study is limited by the variety of data. To obtain a reliable system for OR insights, more specialisms, such as orthopedics and vascular surgery, should be included. The addition of robot-assisted surgery, as is demonstrated in related work [6], is an essential step as well. Preparation and docking of a surgical robot can have a significant impact on operation times, varying with the experience of the OR team [25]. Furthermore, the dataset should be expanded to include multiple ORs at different hospitals and countries. In future work, we want to collect a larger dataset that involves more variety, to build robust models that are not easily confused by unknown scene elements. Additionally, we will explore opportunities to identify more detailed actions in the OR. For example, distinguishing elements in the *surgery* phase, such as preparation, execution of the procedure, and closing the patient, could bring more detailed efficiency insights.

Last, it should be investigated how an automated system for workflow registration can robustly operate in a hospital environment, thereby decreasing rather than increasing the workload on personnel. We envision a low-tech system that consists of a single camera that sends anonymous depth videos over a secure internet connection to a cloud computing service that runs our deep learning algorithm. Subsequently, hospital software could connect to this service to gather the detected workflow steps and efficiency insights. When new hospitals or clinical environments are added, it is likely that the deep learning algorithm will need to be retrained on local data. As our dataset includes 21 procedures for training the algorithm, we expect retraining to require a similar amount of data. Potentially, even less data are needed when our model is used for fine-tuning.

In conclusion, we have shown that the recognition of workflow steps in the OR using privacy-preserving depth videos is feasible using state-of-the-art deep learning models.

## Supplementary Information

Below is the link to the electronic supplementary material.

Supplementary file1 (DOCX 19 KB)
