# Supplementary material for: Automated surgical workflow recognition in privacy-preserving depth videos of the operating room
Source: Surg Endosc. 2025 Aug 6;39(9):5948–56. doi: 10.1007/s00464-025-12031-6 (PMC12408783; doi:10.1007/s00464-025-12031-6)
Supplement: Supplementary file 1 — Supplementary file1 (DOCX 19 KB) [file 464_2025_12031_MOESM1_ESM.docx]

**Supplementary material**

*Hyperparameters used during model training*

The spatial models are trained with a relatively shallow architecture: ResNet-18 and ViT B/16. For these two models, we used a learning rate of 1 x 10^-4^, a batch size of four, class weighting, and trained for a single epoch, all found to perform best empirically. As data augmentation, we applied random cropping between 80-100% of the original image size, and added Gaussian noise with a standard deviation of 0.1. For MS-TCN++, we trained for 100 epochs with a learning rate of 5 x 10^-4^, batch size of one, 32-dim hidden state, and three refinement stages. For ASFormer, we used twenty epochs, a learning rate of 5 x 10^-4^, a batch size of one, a 64-dim hidden state, and a decoder with ten attention layers. DiffAct is trained for 100 epochs with a learning rate of 5 x 10^-4^ and a batch size of four, ten encoder, and eight decoder layers.
